# Molecular and serological epidemiology of Japanese encephalitis virus (JEV) in a remote island of western Japan: an implication of JEV migration over the East China Sea

**DOI:** 10.1186/s41182-016-0010-0

**Published:** 2016-05-03

**Authors:** Akira Yoshikawa, Takeshi Nabeshima, Shingo Inoue, Masanobu Agoh, Kouichi Morita

**Affiliations:** Graduate School of Biomedical Sciences, Nagasaki University, 1-12-4 Sakamoto, Nagasaki, Nagasaki 852-8523 Japan; Department of Virology, Institute of Tropical Medicine, Nagasaki University, 1-12-4 Sakamoto, Nagasaki, Nagasaki 852-8523 Japan; Department of Public Health, Nagasaki Prefectural Institute for Environmental Research and Public Health, 2-1306-11 Ikeda, Omura, Nagasaki 856-0026 Japan

**Keywords:** Japanese encephalitis virus, Remote island, Goto, Isahaya, Nagasaki, Maximum-likelihood phylogenetic tree, Subcluster

## Abstract

**Background:**

Japanese encephalitis (JE) is a mosquito-borne infectious disease caused by Japanese encephalitis virus (JEV). About 1–10 cases with severe central nervous system symptoms have been constantly reported every year in Japan. To clarify the mechanism of maintenance of JEV, the present study surveyed pigs for serological evidence of JEV infection and isolated JEV strains from pigs and mosquitoes in Isahaya City (Isahaya) and Goto City (Goto) in the islets of Goto in Nagasaki Prefecture from 2008 to 2014.

**Results:**

The serological survey of pigs showed the increase of IgM sero-positivity against JEV in July or August, and it was maintained until October or November in both Isahaya and Goto every year. There were 47 JEV strains isolated in Nagasaki from 2001 to 2014 including the isolates in this study, and they belonged to genotype 1. Thirty four of the isolated strains were from pigs in Isahaya and were classified under six subclusters (1-A-1, 1-A-2, 1-A-3, 1-A-4, 1-A-5, and 1-A-9). Thirteen strains were isolated from pigs and mosquitoes in Goto and were classified into three subclusters (1-A-5 (2008); 1-A-1 (2009); and 1-A-2). In the subcluster 1-A-2, three different monophyletic subgroups, 1-A-2-2 (2010), 1-A-2-3 (2011), and 1-A-2-1 (2013, 2014), appeared in Goto.

**Conclusions:**

These data strongly suggested that JEV appearance in Goto seems to depend on the frequent introduction of JEV from outside of the island and this pattern is different from what has been observed in subtropical islands in the East China Sea such as Okinawa and Taiwan, where the same populations of JEV (1-A-7 (1998–2008) in Okinawa; genotype 3 (until 2012) in Taiwan) have been maintained for a long period.

## Background

Japanese encephalitis virus (JEV) belongs to the family *Flaviviridae*, genus *Flavivirus*. It has a positive-sense single-stranded RNA genome which consists of approximately 11,000 nucleotides. The genome encodes three structural proteins and seven non-structural proteins [1]. JEV has five genotypes (1, 2, 3, 4, and 5) [2, 3] with genotype 3 being previously dominant in Japan up to 1990; however, genotype 1 took over and has become dominant until now [3–7].

Japanese encephalitis (JE) is caused by JEV infection through a mosquito bite. Most cases are asymptomatic; however, one out of 100 to 1000 case patients develops clinical symptoms such as high fever, headache, and vomiting, and some are severe central nervous system (CNS) symptoms such as convulsion, paralysis, coma, and encephalitis, which eventually lead to a fatal outcome. The mortality rate of patients with CNS symptoms is 20–40 %, and severe sequelae such as paralysis of motor function and cognitive abnormalities may remain in the patient after recovery [8, 9]. In Japan, JE has decreased drastically due to the introduction of JEV vaccination since the 1970s; however, still around 1–10 JE patients with CNS symptoms are constantly reported every year in the western part of Japan including Nagasaki Prefecture.

JEV is maintained among pigs, birds, and mosquitoes and occasionally in humans who are dead end host. Pigs are recognized as amplifier animal and the sources of JEV during their viremic phase. *Culex tritaeniorhynchus* and other *Culex* spp. are major vector mosquitoes for transmission of JEV [3, 9]. In Japan, seroconversion of pigs against JEV has been being monitored since 1960s and has been observed to occur during summer season (June to September) [10, 11]; however, the maintenance mechanism of JEV circulation during winter is not fully elucidated. JEV appears every summer season in Japan, and there are some reports that JEV circulates within Japan throughout the year [12, 13]. Nabeshima et al. [14] suggested that the sources of JEV are not only within Japan but also from continental Asia where many JE patients and fatal cases have been reported [15].

**Fig. 1:**
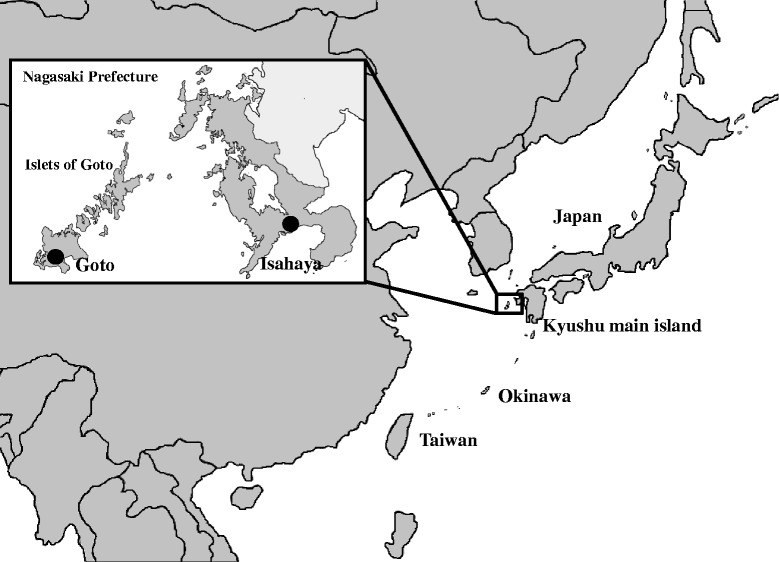
Two observation points in Nagasaki Prefecture for monitoring of JEV. Pig serum samples and mosquitoes were collected in Isahaya and Goto which were indicated by *black solid circles*

The present study aimed to clarify the ecology of JEV in Japan by two approaches: serological survey of swine sera and molecular epidemiological analysis of JEV isolates from pigs and mosquitoes collected in two observation points in Nagasaki Prefecture from 2008 to 2014. Goto City (Goto) in the islets of Goto was selected as an observation point because of its location—a small remote island in western Japan—and the presence of pig farms and wide rice paddy fields which are important factors for JEV maintenance cycle [16]. Another observation point was Isahaya City (Isahaya) located in Kyushu main island (Fig. 1). Many pig farms, wide rice paddy fields, and many wild boars exist in that area. The influence of the geographical uniqueness and environment of these two observation points for the maintenance of JEV was the major interest of this survey.

## Methods

### Swine serum samples

A total of 1740 swine serum samples were collected from approximately 6-month-old pigs at the slaughterhouse in Isahaya City (centered at 32° 50’ 36” N, 130° 3’ 11” E) and several pig farms in Goto City (centered at 32° 41’ 43” N, 128° 49’ 45” E, 100 km west from Nagasaki City, 420 km^2^ land, mean temperature is 17 °C), Nagasaki Prefecture, Japan, from 2009 to 2014 (Fig. 1). A total of 980 and 760 samples were collected, respectively, from Isahaya and Goto as a result of the collection of about 10 to 30 samples from each site every month. Collection of serum samples from pigs to be slaughtered for food consumption was done in accordance with the Guideline on the Conduct of Slaughtering Animals (Ministry of Health, Labour and Welfare) and the Guideline of Animal Welfare (Ministry of the Environment).

### Mosquito samples

From 2008 to 2014, a total of 10,989 mosquitoes were collected near the pig farms in Goto by using CDC light traps. Mosquitoes were identified as to their species, sex, and whether they were engorged with blood or not. There were 8661 *C. tritaeniorhynchus* and *Culex* spp. that were collected and were put together as 474 mosquito pools (about 20 heads per pool). These mosquito pools were mixed with 2.0 mL of Eagle’s minimum essential medium (MEM), homogenized by using electric grinder, and centrifuged at 6142*g* for 10 min at 4 °C. The supernatants were filtered through 0.22-μm pore size membrane filters.

### Virus and cell lines

A total of 47 JEV strains (Table 1) were used in the present study for phylogenetic analysis. Twenty-two strains were obtained from the JEV repository at the Nagasaki Prefectural Institute for Environmental Science and Public Health (NIEP). These were isolated from swine sera that were collected from 2001 to 2007 in Isahaya. Twenty-five strains were isolated in the present study from swine sera and mosquito samples in Isahaya and Goto. These viruses were propagated in the C6/36 mosquito cell line [17] and Vero African Green monkey epithelial kidney cell line (No. JCRB9013; Health Science Research Resources Bank). Both C6/36 cell line and Vero cell line were used for JEV isolation.

**Table 1:** Japanese encephalitis virus isolates in Nagasaki Prefecture

Year	No. of samples	No. of JEV isolates	Collection date	Collection area	Source	Strain name	Subcluster	Accession no.
2001	144 serum samples	9	2001.7.17	Isahaya	Swine serum	JaNP26-01	1-A-9	LC095819
			2001.7.17	Isahaya	Swine serum	JaNP32-01	1-A-9	LC095820
			2001.7.25	Isahaya	Swine serum	JaNP43-01	1-A-9	LC095821
			2001.7.25	Isahaya	Swine serum	JaNP45-01	1-A-9	LC095822
			2001.7.25	Isahaya	Swine serum	JaNP48-01	1-A-3	LC095823
			2001.7.25	Isahaya	Swine serum	JaNP53-01	1-A-9	LC095824
			2001.7.25	Isahaya	Swine serum	JaNP55-01	1-A-9	LC095825
			2001.7.25	Isahaya	Swine serum	JaNP59-01	1-A-9	LC095826
			2001.7.25	Isahaya	Swine serum	JaNP60-01	1-A-9	LC095827
2003	155 serum samples	3	2003.8.4	Isahaya	Swine serum	JaNP59-03	1-A-4	LC095828
			2003.8.4	Isahaya	Swine serum	JaNP76-03	1-A-3	LC095829
			2003.8.12	Isahaya	Swine serum	JaNP84-03	1-A-4	LC095830
2004	133 serum samples	2	2004.8.6	Isahaya	Swine serum	JaNP77-04	1-A-5	LC095831
			2004.8.10	Isahaya	Swine serum	JaNP105-04	1-A-2	LC095832
2005	160 serum samples	5	2005.7.26	Isahaya	Swine serum	JaNP42-05	1-A-2	LC095833
			2005.7.26	Isahaya	Swine serum	JaNP57-05	1-A-2	LC095834
			2005.8.9	Isahaya	Swine serum	JaNP62-05	1-A-2	LC095835
			2005.8.9	Isahaya	Swine serum	JaNP65-05	1-A-2	LC095836
			2005.8.17	Isahaya	Swine serum	JaNP100-05	1-A-2	LC095837
2006	152 serum samples	2	2006.8.8	Isahaya	Swine serum	JaNP69-06	1-A-2	LC095838
			2006.8.8	Isahaya	Swine serum	JaNP70-06	1-A-2	LC095839
2007	80 serum samples	1	2007.7.17	Isahaya	Swine serum	JaNP12-07	1-A-1	LC095840
2008	42 pools	7	2008.8.20	Goto	Mosquitoes	JaNAr01G-08	1-A-5	LC095841
			2008.8.20	Goto	Mosquitoes	JaNAr03G-08	1-A-5	LC095842
			2008.8.20	Goto	Mosquitoes	JaNAr04G-08	1-A-5	LC095843
			2008.8.20	Goto	Mosquitoes	JaNAr11G-08	1-A-5	LC095844
			2008.8.20	Goto	Mosquitoes	JaNAr14G-08	1-A-5	LC095845
			2008.8.20	Goto	Mosquitoes	JaNAr23G-08	1-A-5	LC095846
			2008.8.20	Goto	Mosquitoes	JaNAr33G-08	1-A-5	LC095847
2009	120 serum samples	2	2009.7.28	Isahaya	Swine serum	JaNP22-09	1-A-5	LC095848
			2009.7.28	Isahaya	Swine serum	JaNP25-09	1-A-5	LC095849
	130 serum samples	0		Goto	Swine serum			
	85 pools	1	2009.8.24	Goto	Mosquitoes	JaNAr01G-09	1-A-1	LC095850
2010	170 serum samples	2	2010.8.3	Isahaya	Swine serum	JaNP95-10	1-A-5	LC095851
			2010.8.3	Isahaya	Swine serum	JaNP96-10	1-A-2	LC095852
	130 serum samples	1	2010.8.19	Goto	Swine serum	JaNP84G-10	1-A-2	LC095853
	22 pools	0		Goto	Mosquitoes			
2011	180 serum samples	2	2011.8.5	Isahaya	Swine serum	JaNP92-11	1-A-2	LC095854
			2011.8.5	Isahaya	Swine serum	JaNP97-11	1-A-2	LC095855
	130 serum samples	1	2011.8.18	Goto	Swine serum	JaNP86G-11	1-A-2	LC095856
	58 pools	0		Goto	Mosquitoes			
2012	170 serum samples	5	2012.8.7	Isahaya	Swine serum	JaNP95-12	1-A-2	LC095857
			2012.8.13	Isahaya	Swine serum	JaNP105-12	1-A-2	LC095858
			2012.8.13	Isahaya	Swine serum	JaNP110-12	1-A-2	LC095859
			2012.8.21	Isahaya	Swine serum	JaNP114-12	1-A-2	LC095860
			2012.8.21	Isahaya	Swine serum	JaNP119-12	1-A-2	LC095861
	130 serum samples	0		Goto	Swine serum			
2013	170 serum samples	1	2013.7.23	Isahaya	Swine serum	JaNP90-13	1-A-5	LC095862
	120 serum samples	1	2013.8.13	Goto	Swine serum	JaNP71G-13	1-A-2	LC095863
	116 pools	1	2013.8.17	Goto	Mosquitoes	JaNAr10G-13	1-A-2	LC095864
2014	170 serum samples	0		Isahaya	Swine serum			
	120 serum samples	1	2014.9.22	Goto	Swine serum	JaNP82G-14	1-A-2	LC095865
	151 pools	0		Goto	Mosquitoes			
Total	824 serum samples	22	2001-2008	Isahaya	Swine serum			
	980 serum samples	12	2009-2014	Isahaya	Swine serum			
	760 serum samples	4	2009-2014	Goto	Swine serum			
	474 pools	9	2008-2014	Goto	Mosquitoes			

### Detection of JEV-IgM

To check JEV infection among pigs, in-house JEV IgM-capture ELISA was carried out by following the protocol described by Bundo and Igarashi [18] but with modification. In the present study, anti-pig-IgM antibody (μ-chain specific; BETHYL, Montgomery, TX) was used as catching antibody, JaGAr strain of JEV as the assay antigen, and the mouse monoclonal antibody 6B6C-1 [19] as the horseradish peroxidase-conjugated anti-flavivirus antibody. The optical density (OD) was read at 492 nm by using Multiscan JX (model No.353, Thermolabsystem, Tokyo, Japan). A P/N (positive control or sample serum OD_492_ value/negative control serum OD_492_ value) ratio ≥2.0 was considered positive.

### Virus isolation

Swine sera and filtrated mosquito-homogenate samples were inoculated to the monolayer of Vero cells and C6/36 cells which were kept in maintenance medium (Eagle’s MEM containing 2.0 % heat inactivate-fetal bovine serum) and incubated at 37 and 28 °C, respectively, for 7 days. The infected culture fluid (ICF) was harvested when cytopathic effects (CPE) were observed during two passages and was centrifuged at 3700 *g* for 10 min at 4 °C. The supernatant was then stored at −80 °C until it was used. The presence of virus was verified by reverse transcription-polymerase chain reaction (RT-PCR).

### RT-PCR

RNA was extracted from ICF with QIAamp Viral RNA Mini Kit (Qiagen, Hilden, Germany) according to the manufacturer’s instructions. RT-PCR was carried out using JEV-specific sense primer (JE-NS3-1S; 5′-AGAGCGGGGAAAAAGGTCAT-3′) and anti-sense primer (JE-NS3-4R; 5′-TTTCACGCTCTTTCTACAGT-3′) [20] with SuperScript III One-Step RT-PCR System with Platinum *Taq* (Thermo Fisher Scientific Inc, Waltham, MA). RT-PCR was performed 1 cycle at 53 °C for 30 min and 94 °C for 2 min; 40 cycles at 94 °C for 15 s, 53 °C for 30 s, and 68 °C for 90 s; and 1 cycle at 68 °C for 5 min.

### Sequencing

To complete the nucleotide sequence of the envelope (E) gene of each of the 47 JEV strains isolated in Nagasaki (Table 1), amplified PCR products were purified using QIAquick Gel Extraction Kit (Qiagen, Hilden, Germany) according to the manufacturer’s instructions. The primer extension dideoxy chain termination method was used for direct sequencing of PCR product. For each sequencing reaction, 30–90 ng of purified PCR product was combined with 3.2 pmol of specific primer and BigDye Terminator Cycle Sequencing Ready Reaction Mixture v3.1 containing the four dye-labeled deoxynucleotide terminators (Thermo Fisher Scientific Inc, Waltham, MA). The terminal cycle sequencing parameters were used, as described by the manufacturer. The reaction mixture was column-purified (Performa DTR Gel Filtration Cartridges, Edge BioSystems, Gaithersburg, MD) and kept at 4 °C until it was loaded into the sequencer ABI Prism™ 3130 Genetic Analyzer (Thermo Fisher Scientific Inc, Waltham, MA). The nucleotide sequences of the primers used for the E gene were JE821-840F (5′-GAAAGCCACACGGTATCTCA-3′) and JE2851-2817R (5′-GCAAAGAGAATGCTTTTTCCCCATGCTTTCCAGCC-3′) [14] or JE955f (5′-TGYTGGTCGCTCCGGCTTA-3′) and JE2536r (5′-AAGATGCCACTTCCACAYCTC-3′) [21].

### Phylogenetic analysis

To compare JEV population dynamics in remote islands of East Asia, we obtained 994 strains of genotype 1 JEV E coding region sequences that were available in Genbank. From the data set, we selected 48 strains from Taiwan, 7 strains from Okinawa, and 10 strains from Nagasaki. From the remaining data set, 151 strains were selected by cd-hit version 4.6.1 [22]. The reference sequence of JEV (NC_001437, Strain JaOArS982, genotype 3) and four genotype 3 strains from Nagasaki were used as out-group. We used a total of 268 strains including 47 Nagasaki strains that were sequenced in this work for phylogenetic analysis. Sequence data were aligned using the MAFFT program version 7.245 [23]. The substitution model was selected by jModelTest2 [24]. The phylogenetic tree was constructed by the maximum-likelihood method using the PhyML, version 3.0 [25]. The tree was drawn by FigTree software, version 1.4 (http://tree.bio.ed.ac.uk/software/figtree/).

### DDBJ accession numbers

The DDBJ accession numbers of the JEV Nagasaki strains used in the present study for the construction of the phylogenetic tree as well as date of sample collection, sample collection area in Nagasaki, type of sample, name of strain, and subcluster of strain are listed in Table 1.

## Results

### Serological survey

**Fig. 2:**
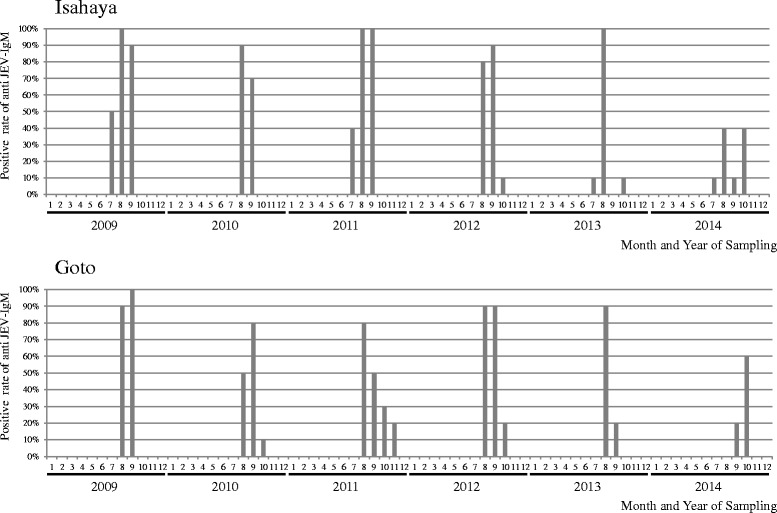
Seasonal trend of Pig IgM positivity against JEV in Isahaya and Goto, 2009 to 2014. Ten to 30 swine serum samples were collected every month in Isahaya and Goto, respectively, and P/N ratio of IgM against JEV using IgM-capture ELISA were calculated every month

During the 6 years (2009 to 2014) of consecutive monitoring of IgM sero-positivity against JEV among 10 to 30 pigs in Isahaya and Goto, a similar pattern was observed almost every year. IgM-positive pigs were detected either in July or August with most of the pigs becoming IgM positive between August and September, and positive detection was sustained up to October or November (Fig. 2). No IgM-positive pig could be detected during winter and spring (December to June) every year during this study. Only a slight difference was observed between Isahaya and Goto, and this was the timing of pig seroconversion which occurred 1 month earlier in Isahaya than in Goto. The seroconversion rates in both Isahaya and Goto in 2014 only reached 40–60 % which was unlike the previous years where the rates were in the range of 80–100 %.

### Virus isolation

During the span of 7 years, 16 strains of JEV were successfully isolated from pigs. Twelve strains were isolated from out of 980 swine serum samples collected in Isahaya, and four strains were from out of 760 swine serum samples in Goto (Table 1). Nine strains of JEV were isolated from out of 474 mosquito pools (*C. tritaeniorhynchus* and other *Culex* spp.) collected in Goto from 2008 to 2014 (Table 1).

### Phylogenetic analysis

**Fig. 3:**
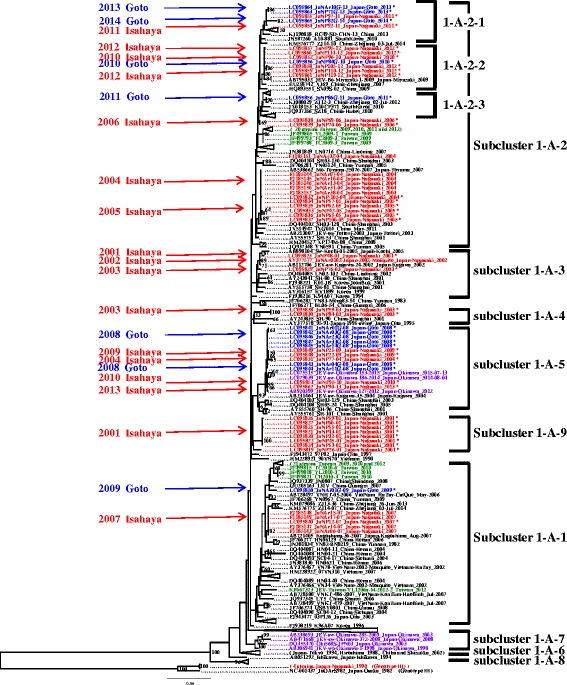
Phylogenetic analysis of the E gene of Japanese encephalitis virus. The phylogenetic tree was constructed by the maximum-likelihood method using the PhyML ver. 3.0 Software. Bootstrap analysis was replicated 1000 times, and bootstrap value (%) greater than 50 % are shown above branches. Genotype 1 JEVs (268 strains) are shown in this figure. Other genotype, genotype 3 (five strains) was used as out-group. Labels of strains conform to the following format: (GenBank or DDBJ accession no.)_(strain name)_(country-region)_(year of isolation). Thirty-four Isahaya strains, 13 Goto strains, 48 Taiwan strains, and 7 Okinawa strains are indicated as *red*, *blue*, *green*, and *purple color*, respectively. JEV strains from China and other areas strains are indicated in *black*

**Fig. 4:**
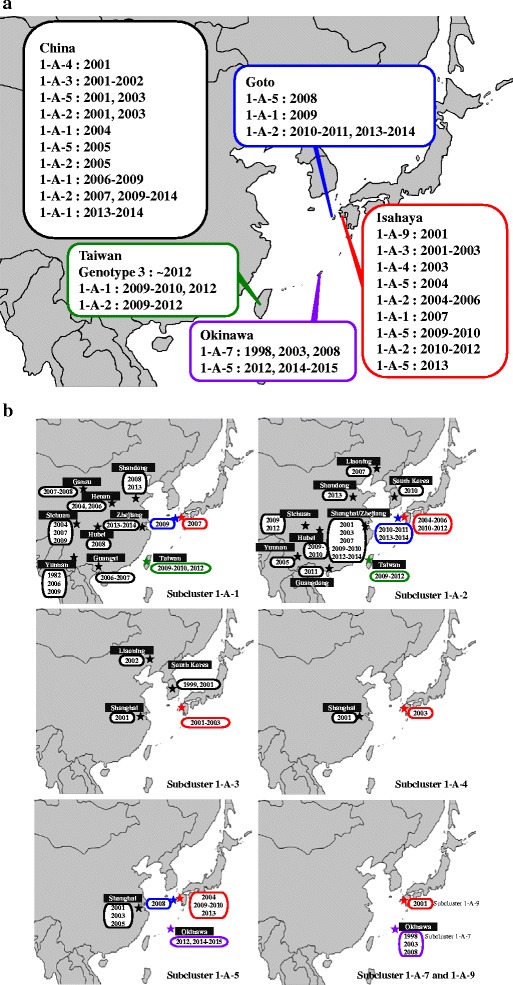
**a** Locations and isolation years of JEVs. Geographical locations of genotype 1 JEVs isolated in Isahaya, Goto, Okinawa, Taiwan, and China are shown in *red*, *blue*, *green*, *purple*, and *black color*, respectively, with subcluster name and year of isolation. **b** Geographical locations of the seven subclusters of JEV genotype 1. Seven subclusters of JEV genotype 1 isolated in Isahaya, Goto, Okinawa, Taiwan, and China are shown in *red*, *blue*, *green*, *purple*, and *black color*, respectively, with year of isolation

Twenty-five strains of JEV isolated in the present study and 22 strains obtained from the JEV repository at the NIEP were analyzed for their E gene sequences (1500 nucleotides). All the 47 strains of JEV Nagasaki isolates belonged to genotype 1. JEV genotype 1 isolates analyzed in the present study were classified into nine subclusters (1-A-1 to 1-A-9, Fig. 3) by following the terminology of Nabeshima et al. [26]. Thirty-four strains isolated from Isahaya were classified into six subclusters which were 1-A-1, 1-A-2, 1-A-3, 1-A-4, 1-A-5, and 1-A-9 (Figs. 3 and 4a, b); and 13 strains isolated from Goto were classified into three subclusters which were 1-A-1, 1-A-2, and 1-A-5. In Goto, subclusters 1-A-1 and 1-A-5 were seen in 2009 and 2008 respectively, and subcluster 1-A-2 has been detected since 2010. The subcluster 1-A-2 contains some monophyletic subgroups. The subgroup 1-A-2-2 appeared in 2010, 1-A-2-3 in 2011, and 1-A-2-1 in 2013 and 2014 (Fig. 3).

## Discussion

In the present study, the results of monthly monitoring of antibody positivity against JEV for six consecutive years from 2009 to 2014 in Isahaya and Goto in Nagasaki Prefecture supported the previous report by the NIEP on pigs sero-surveillance about the pig becoming seropositive during summer season in these areas between July to September (particularly, in the end of July to the beginning of August) [27]. However, their report lacked data on the other three seasons: autumn, winter, and spring (October to June). Thus, the present study clarified pig sero-positivity trend against JEV in all four seasons. During these 6 years, no JEV seropositive pigs were found in both Isahaya and Goto during winter and spring. It suggests that either no transmission of JEV between vector mosquitoes and amplifier animals occurred in these two observation points or it occurred as a very minor incidence which could not be detected by our sampling scale (10~30 heads of pigs per month). The reason of low seroconversion rate in 2014 could be due to unusual climate condition that caused the reduction of sunshine duration to half and the increase in the volume of precipitation twice in August in comparison to the same month in the other years [28].

The genotype shift of JEV from genotype 3 to genotype 1 in Japan and surrounding area was reported before [4, 5, 29, 30], and the current dominant genotype is still genotype 1 [7]. We classified the genotype 1 into nine subclusters (1-A-1 to 1-A-9, Fig. 3). The 47 strains isolated in this study were distributed in six subclusters (1-A-1, 1-A-2, 1-A-3, 1-A-4, 1-A-5, and 1-A-9).

In case of Isahaya, the JEV isolates showed variety of subclusters (1-A-1, 1-A-2, 1-A-3, 1-A-4, 1-A-5, and 1-A-9); furthermore, multiple subclusters of JEV appeared in 2001, 2003, 2004, and 2010. Obara et al. [31] also reported the occasional introduction of minor subcluster "C" (1-A-5 in the present study) in 2007 and the maintenance of major subcluster "A" (1-A-1 in the present study) of JEV in Toyama Prefecture (centered at 36° 41’ 45” N, 137° 12’ 49” E, central north of Japan) from 2005 to 2009 and this pattern was similar to the pattern in Isahaya in the present study.

In Goto, three subclusters were isolated during 7 years (2008 to 2014) in the present study. The subcluster 1-A-5 appeared in 2008, and 1-A-1 appeared in 2009. Since 2010, JEV strains belonging to the subcluster 1-A-2 have been isolated from Goto. In the subcluster 1-A-2, three subgroups, 1-A-2-1, 1-A-2-2, and 1-A-2-3, were distributed not only in Goto but also in China, South Korea, and a main island (Kyushu) of Japan, and each subgroup appeared in Goto in different years (Fig. 3). Replacement of subclusters and subgroups occurred both in Goto and Isahaya, but the subclusters and subgroups that appeared in Isahaya were different from Goto based on the same year of observation. It implies that these subclusters and subgroups were introduced independently to each area, Goto and Isahaya, in 2009 (Isahaya; 1-A-5, Goto; 1-A-1), in 2013 (Isahaya; 1-A-5, Goto; 1-A-2), and in 2011 (Isahaya; 1-A-2-1, Goto; 1-A-2-3).

Goto, Okinawa, and Taiwan are islands in East China Sea. However, in Taiwan, genotype shift from genotype 3 to 1 occurred between 2008 and 2012 [29]. In Okinawa, until 2008, only 1-A-7 JEV strains had been isolated, but in 2012, another subcluster (1-A-5) appeared in Okinawa. These data implied that, in Taiwan and Okinawa, subcluster seemed stable compared with Goto. This could be explained by the mechanism of maintenance of JEV within each area due to the long distance from the mainland China and by the large population of pigs, wide area of rice paddy fields, and subtropical climate in Taiwan and Okinawa as compared to Goto.

It is worthy to note that more frequent replacement of subclusters seems to be occurring in Goto, compared to Taiwan and Okinawa. Nabeshima et al. [14, 26] has proposed the hypothesis for the mechanism of introduction of new subclusters of JEV into Japan from continental Asia by periodical mosquito flying along with the westerlies and overwintering of JEV somewhere in Japan [12, 13]. Otuka [32] reported that planthoppers, *Laodelphax striatellus*, migrate by westerly winds from east China to Japan along the frontal zone, called Bai-u in the Far East. If JEV is also introduced by migration of mosquitoes, it is possible that more frequent introduction occurs in the area around Goto than in Taiwan or Okinawa.

Remote islands provide better setting to investigate viral ecology because migration of terrestrial organisms is restricted and the size of population is small. We expect that chronological surveillance in Goto which had frequent replacement of viral population in contrast to that from subtropical islands such as Okinawa and Taiwan will provide important data to reveal how new arbovirus population migrate, invade, and settle in newly arrived area.

## Conclusions

In conclusion, JEV was being introduced in Goto from outside of the island. The transition patterns of JEV populations in Goto suggested that there were frequent introductions of JEV in Goto island in comparison to Taiwan and Okinawa (subtropical islands in the East China Sea) where the same populations of JEV were being kept for a long time.
